# Supplementary material for: Splice-Junction-Based Mapping of Alternative Isoforms in the Human Proteome
Source: Cell Rep. Author manuscript; Available in PMC 2020 Jan 15. (PMC6961840; doi:10.1016/j.celrep.2019.11.026)
Supplement: 2 [file NIHMS1546469-supplement-2.zip › DF1/PXD006675/PulmonaryVein/PulmonaryVein_25_GEMIN6_GEISALCNLHLAVLSLLEWQDYIYK.pdf]

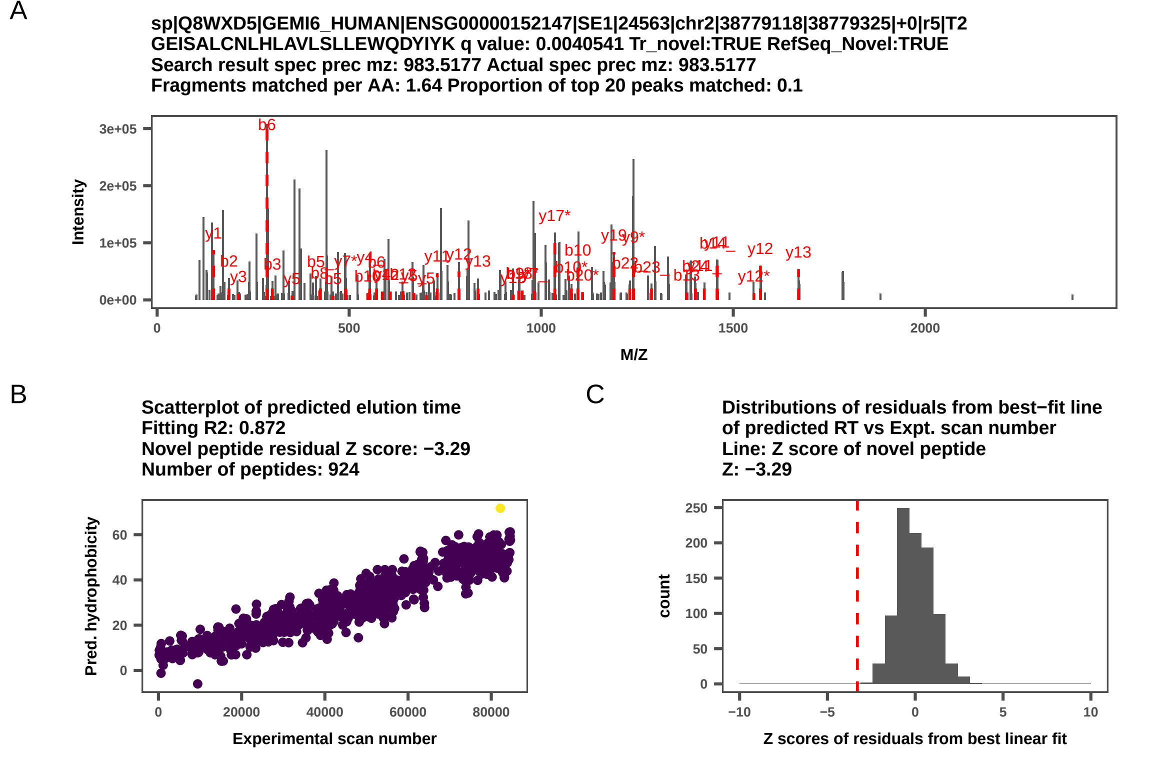
A
sp|Q8WXD5|GEMI6_HUMAN|ENSG00000152147|SE1|24563|chr2|38779118|38779325|+0|r5|T2
GEISALCNLHLAVLSLLEWQDYIYK q value: 0.0040541 Tr_novel:TRUE RefSeq_Novel:TRUE
Search result spec prec mz: 983.5177 Actual spec prec mz: 983.5177
Fragments matched per AA: 1.64 Proportion of top 20 peaks matched: 0.1
Intensity
3e+05
2e+05
1e+05
0e+00
M/Z
0
500
1000
1500
2000
b6
y1
b2
y3
b3
y5
y17*
b10
y12
y13
B
Scatterplot of predicted elution time
Fitting R2: 0.872
Novel peptide residual Z score: −3.29
Number of peptides: 924
Pred. hydrophobicity
60
40
20
0
0
20000
40000
60000
80000
Experimental scan number
C
Distributions of residuals from best−fit line
of predicted RT vs Expt. scan number
Line: Z score of novel peptide
Z: −3.29
count
250
200
150
100
50
0
−10
−5
0
5
10
Z scores of residuals from best linear fit
